# Evaluation of the acceptability of a CD-Rom as a health promotion tool for Inuit in Ottawa

**DOI:** 10.3402/ijch.v72i0.20573

**Published:** 2013-05-23

**Authors:** Kelly E. McShane, Janet K. Smylie, Paul D. Hastings, Conrad Prince, Tungasuvvingat Inuit Family Health Team, Connie Siedule

**Affiliations:** 1 Department of Psychology, Ryerson University, Toronto, ON, Canada; 2 Centre for Research on Inner City Health, St. Michael's Hospital, Toronto, ON, Canada; 3 Dalla Lana School of Public Health, University of Toronto, Toronto, ON, Canada; 4 Center for Mind & Brain, University of California Davis, USA; 5 Tungasuvvingat Inuit Family Health Team, Ottawa, ON, Canada

**Keywords:** Inuit, urban, health promotion, evaluation, community-based research, qualitative, mixed-methods, Elder, Inuktitut, technology, CD-Rom

## Abstract

**Background:**

There are few health promotion tools for urban Inuit, and there is a specific dearth of evaluations on such tools.

**Objective:**

The current study used a community-specific approach in the evaluation of a health promotion tool, based on an urban Inuit community's preferences of health knowledge sources and distribution strategies. In partnership with the Tungasuvvingat Inuit Family Health Team in Ottawa, a CD-Rom was developed featuring an Inuk Elder presenting prenatal health messages in both Inuktitut and English. Also, relevant evaluation materials were developed.

**Design:**

Using a mixed methods approach, 40 participants completed interviews prior to viewing the CD-Rom and participated in a focus group at follow-up. Questionnaires were also completed pre- and post-viewing to assess changes between expectations and reactions in order to document acceptability.

**Results:**

Significant increases were found on satisfaction, acceptability of medium and relevance of content ratings. Qualitative findings also included (a) interest, uncertainty and conditional interest prior to viewing; and (b) positive evaluations of the CD-Rom.

**Conclusions:**

This suggests that CD-Rom technology has the potential for health promotion for urban Inuit, and the community-specific evaluation approach yielded useful information.

Health promotion programmes represent a promising approach to improving the health of Inuit, as messages would be spread effectively among community members, given the strong kinship ties in Inuit communities (1). Although there are some health promotion tools for Inuit, few published reports exist on evaluations of such tools. One recent health promotion program delivered to Inuit in the north examined the effectiveness of a series of media-based programmes for men's wellness, maternity care and youth wellness. Researchers noted that the use of technology was key to accessing such isolated and remote communities (2). For Inuit in southern and urban regions, there is often only limited access to health promotion tools developed in the north, which is of growing concern given the increasing numbers of Inuit living in urban regions (3). Also, the health promotion resources originating in the north were not often deemed applicable to the southern, urban Inuit community. In addition, there are documented differences between the north and the south in health promotion tool needs (4). For instance, it has been reported by Inuit that although access to mainstream resources was easier in the southern and urban regions, these resources were not typically culturally appropriate and were often not available in Inuktitut and contained less connection to Elders. Accordingly, the current study evaluated a new health promotion resource for urban Inuit in Ottawa, designed to maximise the community's previously articulated and existing health information sources and distribution strategies (1).

## Community-specific health promotion tool

This project began with a detailed consultation process with the Inuit community of Ottawa focused on health information sources and distribution strategies (1). Through discussion, 3 main themes emerged regarding existing patterns and articulated preferences: (a) oral–visual learning style of Inuit (medium); (b) pivotal role of Elders (source); and (c) preference for Inuit specific-content (content). With respect to medium, there was a preference for learning about health through oral, direct and face-to-face communication and especially through stories (1). Research from classroom settings with Inuit in the north has found that traditional Inuit learning involves silent observation and listening to other more skilled individuals (5). In light of this, interactive CD-Rom technology would be advantageous because it allows for silent observation and learning, coupled with graphics and recorded messages. In fact, Haggarty and colleagues (6) developed a CD-Rom about suicide education and crisis management for use with Inuit in a small northern community. They reported increased knowledge following use, even though nearly half the sample had never used a computer before, thereby suggesting that this medium would be effective for disseminating knowledge. It should be noted that although a CD-Rom affords opportunities for oral and visual communication, it lacks an ability to capture face-to-face interactions, something important in communication for Inuit. To address this, the current study used a community facilitator to set up and view the CD-Rom with each participant.

Another feature identified was the importance of Elders as sources and distributors of health information. It has been found that Elders could play an important role in public education about teenage pregnancy (4). In Inuit communities in the north, this process of knowledge sharing is natural and effective. For instance, Inuk Elders routinely teach others about the land and the traditional way of life (7). However, with increasing numbers of Inuit moving outside of these northern communities, this process has become difficult. Being able to bridge the gap between the north and south would represent a mechanism to aid in the restoration of this knowledge sharing process. Finally, the third preference reflected a desire for Inuit-specific content, as opposed to pan-Aboriginal content, or content for non-Aboriginal peoples. This is consistent with the previously reported shortage of culturally appropriate Inuktitut resources in Ottawa (4).

## Present study

In collaboration with Tungasuvvingat Inuit Family Health Team (TIFHT), a CD-Rom was developed showing 2 messages about pregnancy and family health presented by an Inuk Elder in Inuktitut. The use of oral and visual media in the CD-Rom represented a match to Inuit learning styles (5) and previously documented preferences for health information distribution (1). In addition, the tool featured an Elder, which addressed both a preference for and familiarity with Elders as knowledge disseminators. Finally, the content was in Inuktitut and from an Inuk Elder, which ensured it was Inuit-specific.

The goal of the present study was to evaluate the acceptability of this guided CD-Rom as a health promotion tool for urban Inuit in Ottawa. Together, the selected medium, source and content were expected to make the CD-Rom an acceptable health promotion resource as it was judged to best represent the articulated patterns and preferences of the community. Given such a match, the acceptability of the tool would be evidenced if after viewing the CD-Rom individuals would (a) share the information with others; (b) consider the medium acceptable; (c) evaluate the source positively; and (d) consider the content helpful.

## Method

This project followed a community-based participatory approach to research and the research team comprised both academic and community researchers. In brief, the project arouse out of long-standing partnership between the community partner and the second author, initially, and the later inclusion of the first author as a student researcher. For this project, the academic researchers and community researchers undertook all aspects of this research together in a collaborative manner and jointly shared decision-making. A more detailed description of the development of the partnership with TIFHT, the participating community, appears elsewhere (1,8,9). The Health Sciences and Science Research Ethics Board at the University of Ottawa approved this study.

### Participants

Individuals were recruited by staff from the Tungasuvvingat Inuit Family Resource Centre (TIFRC) and for the TIFHT in Ottawa, Canada. The Ottawa Inuit community is the largest Inuit community outside of Nunavut, with a population estimate of 725 Inuit residing in the Ottawa-Gatineau area (4) at the time of the study. However, recent research has documented a significant under-participation in the census, suggesting that the actual population of Inuit residing in Ottawa is much greater (10).

The community research team members, who were Inuit community members and front-line health workers, initially identified potential participants. Identified individuals were provided with a description of the study and were then invited to participate if they were interested (all those contacted agreed to participate). Forty adults were recruited to participate in the study (M=39.20 years; SD=12.32 years). This sample included 9 males and 31 females. One participant did not complete the initial interview and another participant did not complete the initial questionnaire. Four participants did not complete the post-CD-Rom evaluation. Three of the 4 were no longer interested in participating in the study, and 1 returned to live in the north.

### Materials

**Fig. 1:**
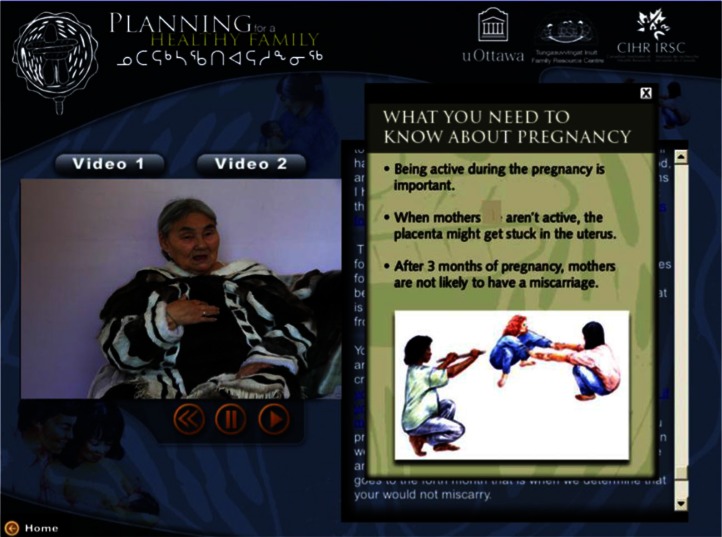
Screen shot of message 1: Summary slide view (English).

**Fig. 2:**
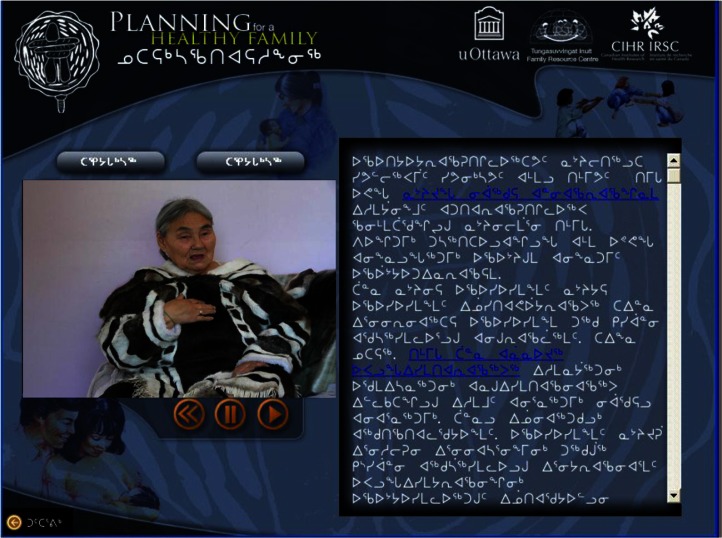
Screen shot of message 2: Video view (Inuktitut).

The CD-Rom was developed in consultation with 2 Elders (from the north) and the academic and community research team. Video segments were selected from a series of brief teachings provided by an Elder on midwifery and prenatal care, based on her >60 years of practice as a midwife. The 2 selected teachings focused on (a) pregnancy and labour and (b) how to support mothers during pregnancy. The Elder believed that it would be of benefit to most members of the Inuit community in Ottawa, whether they were expectant parents themselves or grandparents. The criteria for message selection included clarity and coherence, cultural relevance and consistency with current recommendations regarding the clinical management of pregnancy with respect to physical activity (11). The CD-Rom, entitled, *Planning for a Healthy Family*, contained 2 segments, each with a teaching and text, and a set of summary slides containing major themes and illustrations (see Figs. 1 and 2 for screen shots of the CD-Rom). For the purposes of this study, the CD-Rom was intended to be used by Inuit accessing services, with assistance from the community facilitator (programme staff). In general, this CD-Rom could be used by individuals alone (expectant parents, adults, teens, etc.) or by programme staff during any regular programming. The CD-Rom offered users the choice between Inuktitut and English. Seventeen individuals viewed the CD-Rom in both English and Inuktitut, 14 viewed the CD-Rom in English and 9 viewed the CD-Rom in Inuktitut.

**Fig. 3:**
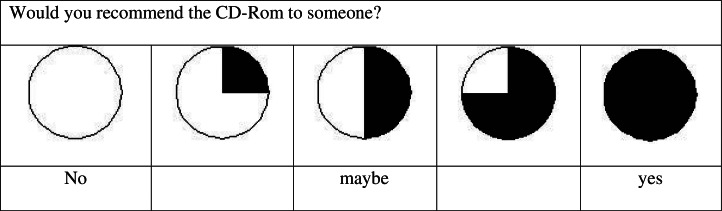
Sample of rating scale questionnaire.

The academic research team, in consultation with the community research team, developed evaluation materials. Open-ended questions were direct and succinct (e.g. “Tell me what it will be ….” vs. “What do you think it will be ….”). In the questionnaire, comparison questions were avoided as they would have required changing the scale anchors, and this was believed by the community research team to create confusion for respondents. For scale anchors, 5 pie charts were used with 3 anchors (“no”, “maybe” and “yes”). No mid point anchors were used to denote “2” and “4” because in Inuktitut, ratings such as “a little” and “a bit” do not exist. See Fig. 3 for a sample of the questionnaire rating system.

The questionnaire items assessed 4 construct areas (a) satisfaction; (b) medium; (c) source; and (d) content. The constructs (b–d) map onto the 3 articulated preferences of the community. Two sets of questionnaires were developed (see Table I); the first to assess expectations (*Pre*) and the second to assess reactions to viewing the CD-Rom (*Post*). Using a pre–post design, the goal was to assess whether post-ratings would be higher than expectations, which would also account for any existing high expectations that could influence results if post-only data had been used.

**Table I:** Questionnaire items and corresponding category outcome

Item number	Category	Item wording (pre)	Item wording (post)
1	Satisfaction	Would you recommend the CD-Rom to someone?	Will you recommend the CD-Rom to someone?
2	Satisfaction	Do you think you'll learn something from the CD-Rom?	Did you learn something from the CD-Rom?
3	Satisfaction	Would you share the information with someone?	Did you share the information with someone?
4	Content	Do you think the message will make sense to you?	Did the message make sense to you?
5	Content	Will the message be helpful for you and your family?	Was the message be helpful for you and your family?
6	Content	Do you think the Inuit-specific information will be useful?	Was the Inuit-specific information useful?
7	Source	Will it be good that the information comes from an Elder?	Was it good that the information came from an Elder?
8	Source	Will it be good that the information comes from Inuit?	Was it good that the information came from an Inuk?
9	Medium	Will it be helpful to have the community facilitator show you the CD-Rom?	Was it helpful to have the community facilitator show you the CD-Rom?
10	Medium	Will the CD-Rom be like talking to someone?	Was the CD-Rom like talking to someone?
11	Medium	Will the CD-Rom be like reading a brochure?	Was the CD-Rom like reading a brochure?

For qualitative data collection, interviews were conducted prior to viewing the CD-Rom, and a focus group was conducted at follow-up (approximately 3 months after viewing the CD-Rom). The interview items were used to explore participants’ expectations about the CD-Rom (e.g. “Tell me what you think it will be like to use the CD-Rom”) and participants’ experience with the CD-Rom (e.g. “How was the CD-Rom different than what you expected?”) and evaluations of features of the CD-Rom (e.g. “What did you like the most about the CD-Rom?”). Questions at the focus group examined how the CD-Rom as a health promotion tool compared to other tools and explored suggestions for changes (e.g. “How does the CD-Rom compare to other ways of sharing health information?”). All materials were translated into Inuktitut prior to commencing the data collection. In addition, an interpreter was used to translate questions during the focus group.

### Procedure

Interested individuals were identified by community research team members and were then provided with a description of the study. If they were interested, participants were presented with a consent form in English and were provided with a description of the study and the consent process in Inuktitut. At the first data collection point, participants answered the interview questions regarding their expectations of using the CD-Rom and completed a questionnaire. About 1 month later (*Post*), participants viewed the CD-Rom with the assistance of a community facilitator. Afterwards, all participants completed a questionnaire. A final focus group was conducted 3 months following the viewing of the CD-Rom, where a subsample of the participants (n=20) participated in a focus group (*Follow-up*).

Participants were given the choice of conducting the interview in Inuktitut or English, as well as a choice for completing the questionnaire (self-report or read in English or Inuktitut by the community researcher). Twenty-seven individuals completed the interview and questionnaire in Inuktitut and 13 completed them in English.

Interviews were all recorded, transcribed and translated for coding purposes. Data analysis used a mixed iterative editorial and organisational approach (12). This approach was also used in a previous study by the research team examining health information processes in the Inuit community in Ottawa (1,9). Initially, the coding was completed by 2 trained academic researchers who listed the most striking themes. A meeting was held between the 2 coders and the community research team to discuss and adapt themes. Following this, the 2 academic researchers took these preliminary themes and developed an initial codebook that was adapted upon a more detailed analysis of the transcripts. Once completed, a finalised codebook listing themes was created. The principal coder coded all transcripts and the reliability coder coded 20 of the transcripts. Inter-rater agreement was calculated using intra-class correlations and was found to range from r=0.62 to 0.89. Disagreements were discussed until consensus was reached between the 2 coders.

## Results

### Preliminary analyses

In order to assess for any differences in ratings as a result of language, mixed multivariate analysis of variance (MANOVAs) were performed, with time and outcome category as within subjects variables and language as a between subjects variable. Four MANOVAs were performed, 1 for each outcome category: (a) satisfaction (items 2, 3, and 4); (b) medium (items 10, 11, and 12); (c) content (items 5, 6, and 7); and (d) source (items 8 and 9). The MANOVA for medium revealed a significant main effect for language, based on Wilks’ criteria, F(3, 29)=7.71, p<0.01, partial η^2^=0.44, but no language by time interaction, F(3, 29)=1.37, *ns*, partial η^2^=0.12. Inspection of the specific dependent variables showed a significant language effect for item 11 (CD-Rom like talking to someone), F(1, 31)=27.64, p<0.01, partial η^2^=0.30, and for item 12 (CD-Rom like reading a brochure), F(1, 31)=34.91, p<0.001, partial η^2^=0.38. For both items 11 and 12, ratings completed by Inuktitut-speakers (M=3.90 and 4.40, SD=1.37 and 0.91, respectively) were higher than ratings completed by English-speakers (M=2.93 and 3.12, SD=1.08 and 1.20, respectively). Therefore, Inuktitut-speakers expected and found their interactions with the CD-Rom to be higher in similarity to both talking to someone and reading a brochure, compared to English-speakers. No other MANOVA was significant. As the language by time interaction was not significant, it was not necessary to control for language in the quantitative analyses assessing changes in ratings.

### Quantitative analyses

Multivariate analyses of variance (MANOVAs) were performed to examine differences between pre- and post-ratings. As there were multiple items measuring the same construct, a MANOVA was selected to assess differences between pre and post for each of the 4 constructs. Four MANOVAs were performed, with time and outcome category as independent variables, 1 for each outcome category: (a) satisfaction (items 2, 3, and 4); (b) medium (items 10, 11, and 12); (c) content (items 5, 6, and 7); and (d) source (items 8 and 9). Descriptive statistics are presented in Table II.

**Table II:** Descriptive statistics of outcome measures

		Pre		Post	
			
Outcome category	Item	M	SD	M	SD
Satisfaction (N=31)	2	4.26	1.18	4.84	0.64
	3	4.71	0.64	4.90	0.40
	4	4.90	0.30	4.84	0.52
Medium (N=33)	10	4.61	0.79	4.79	0.78
	11	3.33	1.49	4.21	1.32
	12	3.85	1.18	3.91	1.68
Source (N=34)	8	4.94	0.34	5.00	0
	9	4.94	0.34	5.00	0
Content (N=31)	5	4.42	0.89	4.90	0.40
	6	4.40	0.84	4.61	0.96
	7	4.87	0.56	4.81	0.65

**Fig. 4:**
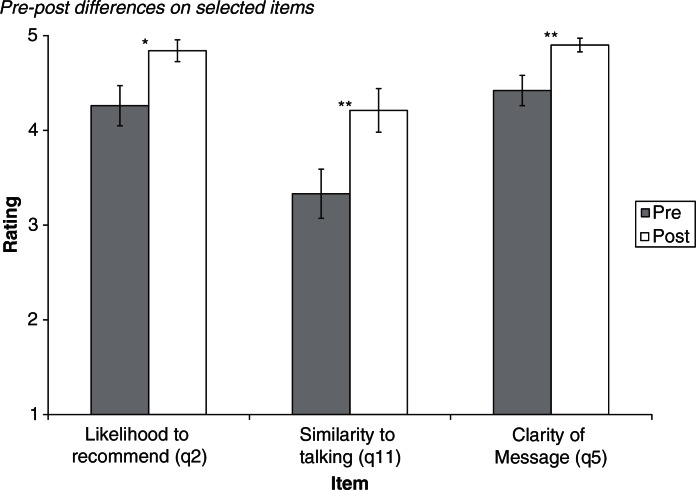
Pre–post differences on selected items. Note: *p < 0.05; **p < 0.01.

The overall multivariate test for a time effect on the 3 satisfaction dependent variables, based on Wilks’ criteria, was significant, F(3, 28)=4.71, p<0.01, partial η^2^=0.34. Inspection of the specific dependent variables revealed a significant effect for time for item 2 (recommend to someone), F(1, 30)=5.09, p<0.05, partial η^2^=0.15. Ratings of likelihood to recommend the CD-Rom were higher after having viewed it (see Fig. 4).

The overall multivariate test for a time effect on the 3 medium dependent variables, based on Wilks’ criteria, was significant, F(3, 30)=3.84, p<0.05, partial η^2^=0.28. Inspection of the specific dependent variables revealed a significant effect for time for item 11 (CD-Rom similar to talking to someone), F(1, 32)=11.73, p<0.01, partial η^2^=0.27. Ratings of similarity to talking to someone were higher after having viewed it (see Figure 4).

The overall multivariate test for a time effect on the 3 content dependent variables, based on Wilks’ criteria, was marginally significant, F(3, 28)=2.67, p<0.10, partial η^2^=0.22. Inspection of the specific dependent variables revealed a significant effect for time for item 5 (message clarity), F(1, 30)=7.85, p<0.01, partial η^2^=0.21. Ratings of how much sense the message made were higher after having viewed it (see Fig. 4).

The overall multivariate test for a time effect on the 2 source-dependent variables, based on Wilks’ criteria, was not significant, F(1, 33)=1.00, p>0.10, partial η^2^=0.03.

### Qualitative findings

To supplement and clarify the quantitative results, the interviews conducted prior to viewing the CD-Rom were coded for themes. Three main themes emerged were interest, uncertainty and conditional interest. Individuals expressed an *interest* in the CD-Rom, including the technological aspects of the tool, the cultural content (language and Elder), as well as the information contained in the tool. They also spoke about their intent to use the information and the CD-Rom, and some requested to have a copy of the CD-Rom at the end of the study. One participant commented, “I know I'm going to want one1, I know it's going to help a lot of people”.

Individuals expressed a general *uncertainty* about the CD-Rom. Some expressed concern specifically about technology (e.g. ability to use computer, navigate tool). Others mentioned concern about the content, for instance expressing concern about the ability to understand the message and language. Some individuals expressed an interest in the CD-Rom and its information if a certain *condition* was met; for example, someone commented it would be good “If I'm doing it with someone”. Other criteria included ease of technology, clarity of content and cultural-appropriateness of message.

Qualitative results of the focus group focused on the preferred aspects of the CD-Rom and suggestions for future work. When community members were asked which feature they preferred the most about the CD-Rom, they were divided between “All” and “the Elder”. One person described the effect of the Elder's voice.

I found <the Elder> to be, her voice is very soothing she is very low key, it's very easy to listen to her, and her, she has a very benign, not a preachy kind of demeanor, she's passing on information, and that, there just something very easy to listen.

Some people commented about liking the traditional information and the fact that the CD-Rom was set up with 2 focused messages. The wholistic nature of the messages was again mentioned.

She like <s> the idea of talking about pregnancy from a wholistic point of view with the help of the mother and the help of the father and that is something that we need talk more <about> as a family value.

Others made comments about the visual information. For instance, 1 participant stated:

The visuals good too because even when you have it in English you can still read her body language.

When asked what they would change about the CD-Rom, community members stated that the message was too short. Individuals were divided about whether there should be a few long messages or whether there should be many short messages. One person commented about the English translation: “As an urban Inuit I thought the English translation was a bit repetitive, so maybe have it ah, more edited a bit on the translation”. There was a request for additional topics, akin to a series that the Elder could talk about. There were also a few comments about enhancing the technology by allowing for a full screen viewing and mouse-over pop-up features for the graphics.

When participants were asked about how the CD-Rom compared to other ways of sharing information, 1 individual commented that “It's not just something you can just pick up and throw away”. One comment was made that it was not as good as face-to-face, but it was better than paper-based information. There was a discussion around accessibility, given that a computer is needed. However, others pointed out that a computer was available at the various community based services.

## Discussion

Few health promotion tools have been evaluated for use with Inuit, and none that we are aware of have been developed and evaluated for urban Inuit specifically. This study evaluated the acceptability of a guided CD-Rom as a health promotion tool for urban Inuit in Ottawa. Overall, the quantitative analyses largely supported predictions regarding the feasibility of such a tool. Although initial expectations were highly positive, overall increases in satisfaction ratings were observed, in particular when assessing the likelihood to recommend the tool to another. This is particularly meaningful as it suggests that this tool represents a viable medium for information sharing. It is also very encouraging that even though the CD-Rom focused specifically on pregnancy and prenatal care, a broad range of community members would recommend the tool to others. This might be because prenatal teachings are important to all members of the community, regardless of their stage of life. In addition, the messages provided participants the opportunity to gain more knowledge about traditional Inuit health. As 1 person commented, “I'm half Inuit and I don't know a lot of our traditional ways so this will give me a good idea of the traditional ways”. Furthermore, given that the Inuk Elder was from the north, this provided community members with an opportunity to learn new Inuktitut words. Given that the Inuktitut language has changed over the years, it is extremely important that traditional Inuktitut words survive (13). The CD-Rom provided an excellent tool to aid in the retention of both language and culture. The desire to retain culture and language has previously been documented with urban Inuit (1), as well as Inuit in the north (7,14). Likewise, numerous research articles comment on the specific role Inuit Elders play in promoting knowledge, language and culture (14).

Ratings of similarity between CD-Rom and engaging in a conversation with someone increased from pre to post. This is significant because face-to-face communication has been identified as 1 of the key methods of sharing health information in the urban Inuit community. Furthermore, community members have previously commented about the difficulty in accessing Elders (1). If the CD-Rom is similar enough to talking to someone, it could represent a feasible and cost-effective method to link Elders living in the North with urban Inuit populations. In addition, the follow-up focus group further highlighted the importance of the Elder in the success of the CD-Rom. This has similarly been observed by Stillwater and colleagues (15) in their work on health promotion for cervical cancer with Alaskan women. Overall, these results suggest that health promotion tools for urban Inuit will be most successful if Elders are involved in the project.

With respect to the technology, it appears that the CD-Rom was easy to navigate. However, it is possible that having a community facilitator present during the viewing of the CD-Rom was responsible for the acceptance, as it is unclear if individuals would have regarded the CD-Rom as easy to use if they had been using a computer on their own, potentially in their home. Also, there remains the issue of accessibility to a computer with which to view the CD-Rom. The follow-up focus group captured the concern that some community members felt about being able to access a computer. For this study, community members viewed the CD-Rom on 1 of 2 office computers that are normally reserved for employees. Alternatively, if DVD technology was used, it is possible that there would be greater accessibility.

Overall, the evaluation of the CD-Rom was positive and indicates that CD-Rom technology has strong potential as a medium for health information sharing in this urban Inuit community. Additional research is needed to assess the impact that the information has on participants’ experiences with pregnancy and prenatal care, and whether attitudes or behaviours changed as a result of having viewed the CD-Rom. The technology itself represents a practical and cost-effective way to connect community members in urban areas with Elders in the north. During the course of this research project, community members expressed a desire for additional health promotion materials, and several participants articulated an interest in materials about parenting young children.
